# HIV-1 reverse transcriptase inhibitory activity of *Aerva lanata* plant extracts

**DOI:** 10.1186/1471-2334-14-S3-P12

**Published:** 2014-05-27

**Authors:** Rajendra Prasad Gujjeti, Sainath Namthabad, Estari Mamidala

**Affiliations:** 1 Infectious Diseases Research Lab, Department of Zoology, Kakatiya University, Warangal, Andhra Pradesh, India

## Background

HIV-1 reverse transcriptase (HIV-1 RT) is an essential enzyme for the replication cycle of HIV. HIV-1 RT inhibitors have been extensively investigated for their anti-HIV properties. However, emergence of HIV drug resistance and side effects are the main reasons for failure of anti-HIV therapy. The aim of the present study was to evaluate the HIV reverse transcriptase inhibitory activity of *Aerva lanata* plant extracts.

## Methods

Extracts were prepared from dried roots in different solvents. Peripheral Blood Mononuclear Cells (PBMCs) were isolated from healthy donors by ficoll-hypaque density gradient centrifugation method. A toxicity study was performed on all crude extracts among PBMCs by MTT assay. HIV-1 RT inhibition activity of the all solvent extracts of *A. lanata* was determined by a HIV-1 Reverse Transcriptase activity assay.

## Results

All the five solvent crude extracts of *A. lanata* were non cytotoxic up to 0.75 mg/mL concentration in PBMCs. Chloroform and methanol extracts shows highest inhibition of recombinant HIV RT (91% and 89% respectively) at 1 mg/mL concentration. This strong inhibitory effect was confirmed by their IC^50^. More than 50% inhibition of HIV RT shows from 0.03 to 1 mg/mL concentrations of all extracts.

## Conclusion

Experimental results thus suggested that the *A. lanata* plant extracts which have been tested in the present study exert their anti-HIV activity via inhibition of HIV Reverse Transcriptase activity. However, in order to assess the usefulness of this herb, it is necessary to isolate the active principle (s) from the crude extracts.

